# Proteins related to ictogenesis and seizure clustering in chronic epilepsy

**DOI:** 10.1038/s41598-021-00956-6

**Published:** 2021-11-02

**Authors:** Woo-Jin Lee, Jangsup Moon, Jung-Ah Lim, Daejong Jeon, Jung-Suk Yoo, Dong-Kyu Park, Dohyun Han, Soon-Tae Lee, Keun-Hwa Jung, Kyung-Il Park, Sang Kun Lee, Kon Chu

**Affiliations:** 1 Department of Neurology, Seoul National University Hospital, 101, Daehak-ro, Jongno-gu, Seoul, 03080 South Korea; 2 Program in Neuroscience, Neuroscience Research Institute of SNUMRC, Seoul National University College of Medicine, Seoul, South Korea; 3 Department of Genomic Medicine, Seoul National University Hospital, Seoul, South Korea; 4 Department of Neurology, Cham Joeun Hospital, Gwangju, South Korea; 5 Advanced Neural Technologies, Seoul, South Korea; 6 Proteomics Core Facility, Biomedical Research Institute, Seoul National University Hospital, Seoul, South Korea; 7 Department of Neurology, Seoul National University Healthcare System Gangnam Center, Seoul, South Korea

**Keywords:** Neurology, Neurological disorders, Proteomics

## Abstract

Seizure clustering is a common phenomenon in epilepsy. Protein expression profiles during a seizure cluster might reflect the pathomechanism underlying ictogenesis. We performed proteomic analyses to identify proteins with a specific temporal expression pattern in cluster phases and to demonstrate their potential pathomechanistic role. Pilocarpine epilepsy model mice with confirmed cluster pattern of spontaneous recurrent seizures by long-term video-electroencpehalography were sacrificed at the onset, peak, or end of a seizure cluster or in the seizure-free period. Proteomic analysis was performed in the hippocampus and the cortex. Differentially expressed proteins (DEPs) were identified and classified according to their temporal expression pattern. Among the five hippocampal (HC)-DEP classes, HC-class 1 (66 DEPs) represented disrupted cell homeostasis due to clustered seizures, HC-class 2 (63 DEPs) cluster-onset downregulated processes, HC-class 3 (42 DEPs) cluster-onset upregulated processes, and HC-class 4 (103 DEPs) consequences of clustered seizures. Especially, DEPs in HC-class 3 were hippocampus-specific and involved in axonogenesis, synaptic vesicle assembly, and neuronal projection, indicating their pathomechanistic roles in ictogenesis. Key proteins in HC-class 3 were highly interconnected and abundantly involved in those biological processes. This study described the seizure cluster-associated spatiotemporal regulation of protein expression. HC-class 3 provides insights regarding ictogenesis-related processes.

## Introduction

Epilepsy is a chronic disorder of recurrent unprovoked seizures^1^. Seizure clustering, which refers to a characteristic of seizure attacks that tend to be closely grouped with short intervals^2–4^, is a common phenomenon in patients with epilepsy, with a frequency reported from 7 to 83%, and it has also been observed in several animal models of chronic epilepsy^2–5^. We have recently observed a robust clustering pattern of spontaneous recurrent seizures (SRS) in a mouse pilocarpine model that presented 97% of all seizures within a cluster period and demonstrated that seizure clusters also have a highly cyclic pattern with steady and long inter-cluster intervals^5^.

Two major pathomechanistic components constitute the seizure clustering phenomenon, the mechanism of seizure clustering and the mechanism of cyclic seizure occurrence. The former is widely regarded as resulting from proconvulsive effects induced by an index seizure, whereas the latter comprises the preictal processes and seizure initiation mechanisms that contribute to the transition to seizure, called ictogenesis^6^. These findings imply that the common pathomechanism of seizure clustering might be dynamic within a cluster^7^, and the cyclic pattern of seizure clusters might serve as a good model to investigate the dynamic mechanism of ictogenesis. Considering that seizure clustering is associated with intractable epilepsy and a higher risk of seizure-related complications^3,4^, understanding the cellular, synaptic, and neuronal network components of seizure clustering mechanisms and elucidating the key molecules might be crucial to find a novel strategy to reduce the morbidity of patients with intractable epilepsy.

Complex regulatory networks among mRNAs, micro RNAs, long non-coding RNAs, circular RNAs, and proteins in epilepsy pathogenesis have been increasingly elucidated, based on genomic, epigenomic, and proteomic analyses^8–14^. Given that the dynamic changes in those regulatory networks might also be fundamentally involved in the periodic occurrence of seizure clusters, proteomic analysis has the advantage that it addresses the most downstream target of those regulatory networks. This enables the evaluation of highly dynamic and location-specific cellular processes and reflects comprehensively the effects of those complex regulatory networks^15^.

In this study, we hypothesized that changes in protein expression profiles in different phases of a seizure cluster might reflect the key pathomechanism underlying the development and termination of seizure clusters. To identify proteins with a specific temporal expression pattern during a seizure cluster and to demonstrate their potential pathomechanistic roles, we performed brain proteomic analyses in a murine pilocarpine epilepsy model.

## Methods

### Generation of the epilepsy model and confirmation of seizure clustering

The pilocarpine epilepsy model was generated according to the procedures described in our previous studies that used the same model^10–14,16,17^. In brief, a single intraperitoneal injection of pilocarpine (400 mg/kg; Sigma, St. Louis, MO, USA) was administered in 70 five-week-old male C57BL/6 mice to induce SE. To minimize systemic effects of the induced muscarinic activation, methyl-scopolamine (1 mg/kg; Sigma) was intraperitoneally injected 30 min prior to the pilocarpine injection. SE onset was defined as the development of continuous tonic–clonic seizures (Racine stage ≥ 4). After 40 min from the onset of SE, diazepam (5 mg/kg) was administered intraperitoneally to terminate the convulsive SE^10–14,16,17^.

Long-term video-EEG monitoring was performed according to the protocols described in our previous reports^5^. EEG electrodes were implanted ≥ 7 days before the initiation of EEG recordings to allow the stabilization of the mouse. Ketamine (90 mg/kg) and xylazine (40 mg/kg) were intraperitoneally injected, EEG electrodes (stainless steel, 1.0 mm in diameter) were implanted at ± 1.8 mm anterioposterior and ± 2.1 mm mediolateral from bregma, and the ground electrode was placed in the cerebellum, using a stereotaxic apparatus (Kopf Instruments, Tujunga, CA, USA). EEGs were recorded using a digital system (Comet XL, Astro-Med, Warwick, RI, USA) with parameters as follows: amplification ratio of × 1200; band-pass filtering between 0.1‒70 Hz; and sampling rate of 400 Hz. EEG data were analyzed using PSG Twin 4.3 (Astro-Med) software. EEG recordings were reviewed by visual inspection^5^.

SRS were defined as a more than two-fold EEG amplitude increment from baseline with repetitive spikes, accompanied by behavioral seizures according to a Racine stage ≥ 4, with durations of ≥ 10 s^5^. The main reason for designating a behavioral seizure severity criteria was to increase the specificity of seizure determination by minimizing the false-positive seizure detection from EEG analysis due to movement-related artifacts^5^. After ≥ 15 weeks from the induction of SE, a seizure cluster was defined as follows: (1) a seizure frequency of ≥ 1 per day for at least three consecutive days and (2) at least five total seizures during the cluster period^5^. The mice were sacrificed after obtaining the video-EEG monitoring data of ≥ 2 full consecutive cycles of seizure clusters.

### Tissue preparation

**Figure 1:**
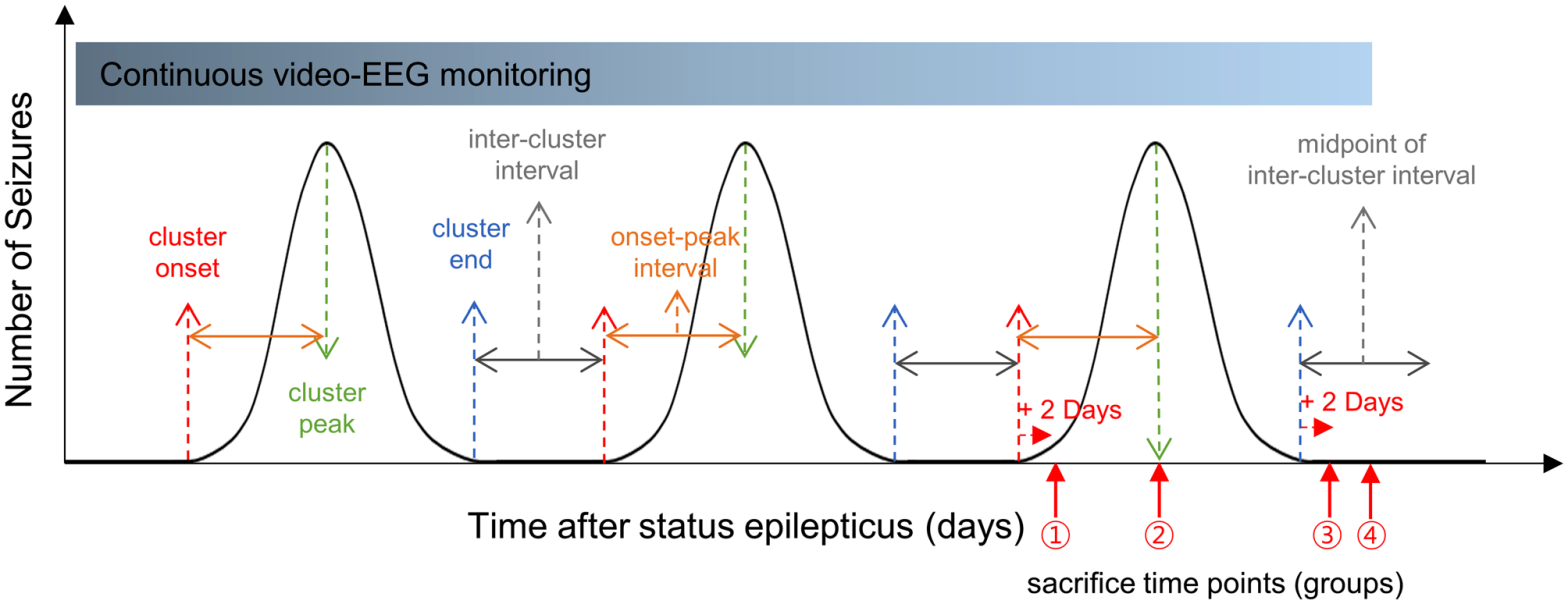
Brain tissue sampling time points during a seizure cluster. Prior to euthanasia, all animals were monitored with video-electroencephalography (video-EEG) for ≥ 2 full consecutive cycles of seizure clusters. Onset (red dotted arrows), peak (green dotted lined), and end (blue dotted lines) of each seizure cluster were checked. The interval between the onset and the peak of a seizure cluster and the interval between two adjacent clustered were measured for each animal. Based on that, the animals were divided into four groups according to the different sacrifice time points, marked by red arrows and circled numbers as follows: group 1 (seizure cluster onset), group 2 (seizure cluster peak), group 3 (seizure cluster end), and group 4 (mid seizure-free).

In our previous study, we observed that the mean inter-cluster seizure-free period was 16.3 ± 6.8 days, and the intervals from cluster onset to the peak seizure frequency day and from the peak seizure frequency day to the end of the cluster were 3.4 ± 1.9 days and 1.2 ± 1.1 days, respectively^5^. Therefore, the animals were divided into four groups according to the different sacrifice time points; onset of the seizure cluster (group 1), peak of the seizure cluster (group 2), end of the seizure cluster (group 3), and mid seizure-free period (group 4). When a mouse had seizures on two consecutive days, the mouse was sacrificed on the second day and classified as group 1. When the number of SRS per day was increasing, the mouse was sacrificed at the estimated day of the peak seizure frequency according to the prior seizure cluster and was classified as group 2. If a mouse did not have a seizure after a seizure cluster period, the mouse was sacrificed at the second seizure-free day and was classified as group 3. During seizure-free periods, some mice were sacrificed at the midpoint of the presumed seizure-free period according to prior clusters, and these mice were classified as group 4 (Fig. 1). Mice were euthanized by carbon dioxide inhalation and brains were immediately removed. Hippocampus and cortex were separated and stored at − 80 °C^10–14,16,17^.

### Sample preparation

Hippocampus and cortex tissues were washed three times with sterilized PBS. Washed tissues were lysed by sonication in 200 ul of lysis buffer (4% SDS, 2 mM TCEP in 0.1 M Tris pH 8.5) for 2 min. For construction of spectral library, BV-2 and HT22 cells were lysed in 100 ul of lysis buffer with 1 min of proved-tip sonication. Protein concentration of tissue and cell lysate was measured using a bicinchoninic acid reducing agent compatibility assay kit (Thermo Fisher Scientific, Waltham, MA, USA)^18,19^.

For preparation of tissue samples, 100 ug of proteins was precipitated overnight at − 20℃ using ice-cold acetone. For spectral library, we performed acetone precipitation at − 20 °C with 300ug of the cell lysate. Protein digestion was performed via the 2-step FASP procedure as described with some modifications^18,19^. Protein pellets were dissolved in SDT buffer (4% SDS, 10 mM TCEP, and 50 mM CAA in 0.1 M Tris pH 8.0) and loaded onto a 30 K Amicon filter (Millipore, Burlington, MA, USA). The buffer exchanges were performed with UA solution (8 M urea in 0.1 M Tris pH 8.5) via centrifugation at 14,000 × g for 15 min. Following the exchange of buffer with 40 mM ammonium bicarbonate (ABC), protein digestion was performed at 37℃ overnight using a trypsin/LysC mixture (Promega, Madison, WI, USA) at a 100:1 protein-to-protease ratio. The digested peptides were collected by centrifugation. After the filter units were washed with 40 mM ABC, second digestion was performed at 37℃ for 2 h using trypsin (enzyme-to-substrate ratio of 1:1000). All resulting peptides were acidified with 10% TFA and desalted using homemade C18-StageTips as described^18,19^. Desalted samples were completely dried with a vacuum dryer and stored at − 80℃.

### Peptide fractionation

StageTip-based, high-pH peptide fractionation was performed as described with some modifications^19^. Peptides obtained from pooled samples were dissolved in 200 ul of loading solution (10 mM ammonium formate solution, pH 10 and 2% acetonitrile) and separated on the reversed-phase tip columns, prepared by packing POROS 20 R2 (Invitrogen, Carlsbad, CA, USA) into a 200-ul yellow tip with C18 Empore disk membranes (3 M, Bracknell, UK) at the bottom. After conditioning of microcolumns with methanol, acetonitrile, and loading buffer, peptides were loaded at pH 10, and 20 fractions were subsequently eluted with buffer solutions, pH 10, containing 5%, 10% 15%, 20%, 25%, 30%, 35%, 40%, 60%, and 80% acetonitrile. To improve the orthogonal fractionation of the RP-RP separation, 20 fractions were combined into six fractions in a noncontiguous manner. The six fractions were dried in a vacuum centrifuge and stored at − 80℃ until liquid chromatograph-tandem mass spectrometer (LC–MS/MS) analysis.

### LC–MS/MS analysis

Liquid Chromatography Mass Spectrometry (LC–MS/MS) analysis was performed using hybrid quadrupole Orbitrap mass spectrometers, Q-exactive plus (Thermo Fisher Scientific) coupled to an Ultimate 3000 RSLC systems (Dionex, Thermo Fisher Scientific) via a nano electrospray source, as described with some modifications^19,20^. Fractionated peptide samples (1ug) were separated on the 2-column setup with a trap column (300 um I.D. × 5 mm, C18 3 um, 100 Å) and an analytical column (50 um I.D. × 50 cm, C18 1.9 um, 100 Å). After the samples were loaded onto the nano LC, a 120-min gradient from 8 to 30% solvent B (100% acetonitrile and 0.1% formic acid) was applied to all samples. The spray voltage was 2.0 kV in the positive ion mode, and the temperature of the heated capillary was set to 320 °C. Mass spectra were acquired in data-dependent mode using a top 15 method. The Orbitrap analyzer scanned precursor ions with a mass range of 300–1650 m/z and a resolution of 70,000 at m/z 200. HCD scans were acquired at a resolution of 17,500 with a normalized collision energy (NCE) of 28. The maximum ion injection time for the survey and MS/MS scans was 25 ms and 50 ms, respectively.

### Data processing

All MS raw files were processed in Maxquant software (ver. 1.5.3.12) built in peptide identification algorithm, Andromeda search engine^21^ to search spectra against the UniprotKB Mouse FASTA database (82,074 entries; version from October 2014) and contaminants. MS/MS searches were performed with the following parameters: Peptides had to have a minimum length of six amino acids to be considered for identification. Carbamidomethylation of cysteine was set as a fixed modification, while oxidation of methionine and protein N-terminal acetylation were set as variable modifications. Enzyme specificity was set to trypsin allowing up to two mis-cleavages. Main and first search tolerance were set 6 ppm and 20 ppm, respectively. The false discovery rate (FDR) for all proteins and peptides of 1% was applied. Retention time of all analyzed samples were linearized in Maxquant by “Match between runs” allowing transfer of identified peptide in absence of sequencing with retention time window of 2 min. All proteins were filtered for common contaminants such as keratins. The MaxLFQ algorithm was used for label-free quantification of proteins^22^.

### Analysis of the differentially expressed proteins

All statistical analyses were performed using Perseus software (http://www.perseus-framework.org)^23^. For the comparison of groups at different time points within a seizure cluster, we first filtered out proteins with less than three quantified values in each group and imputed missing values based on a normal distribution (width = 0.3 and downshift = 1.8). To evaluate the effect of time from SE or the number of seizures on the overall protein expression, correlation analyses between the mean values of normalized protein expression level in the hippocampus and in the cortex with the time from SE or the number of seizures to brain tissue acquisition were performed. Inter-group comparison of normalized protein expression level was also performed. DEPs among the four time points were detected using an ANOVA with a significance level of *P* < 0.05. After DEPs were z-normalized for their abundances, hierarchical clustering was performed according to their expression patterns at the four time points using hierarchical clustering with Euclidean distance. The DEP classes were identified separately in the hippocampus (HC-classes) and in the cortex (CO-classes), and the frequencies of protein overlaps were evaluated for each DEP class in the hippocampus and the cortex.

### Bioinformatics analysis

GO analysis was performed using the DAVID bioinformatics tool^24^. The GO classification was evaluated by Fisher’s exact test to obtain *P* values, which were filtered at a cut-off value of 0.05. For network analysis, protein–protein interactions (PPIs) of DEPs in each class were interrogated based on the STRING database^25^. The PPIs in the network model were visualized using Cytoscape software (version 3.1).

### Ethics approval and consent to participate

All procedures regarding animal care and handling were approved and supervised by the Institutional Animal Care and Use Committee at Seoul National University Hospital, and all experiments were performed in accordance with relevant guidelines and regulations. This study is reported in accordance with ARRIVE guidelines.

## Results

### Overall expression profile of proteins

Among the 70 mice initially included in the experiment, 34 (48.5%) died during the induction of pilocarpine-induced status epilepticus (SE), and 5 (7.1%) mice did not develop SE. Long-term continuous video-electroencephalography (EEG) recordings were performed in the remaining 31 mice. All mice exhibited SRS with seizure clusters. Video-EEG monitoring was discontinued in 16 mice due to dislodged or noisy electrodes during recordings, and the remaining 15 mice were timely sacrificed, 4 mice per group except for group 2 with 3 mice. To have equal numbers in each group, 3 mice each from groups 1, 3, and 4 were selected, according to the better regularity in duration of seizure cluster, number of seizures at the cluster peak, and intervals between the seizure clusters. As the result, 12 mice in total were selected for the proteomic analysis. The mean time period between SE induction and brain tissue acquisition was 231.6 ± 39.4 days (median 235 days, interquartile range [IQR] 193‒265 days, and range 181‒287 days) and the mean duration of video-EEG monitoring was 143.3 ± 57.8 days (median 139 days, IQR 93–198.5 days, range 62–231 days), which were longer than those of our previous study (mean 109.7 ± 20.4 days and mean 53.7 ± 20.4 days, respectively)^5^. The number of monitored seizure clusters before euthanasia was 2.4 ± 0.5 (range 2‒3), with a mean total number of seizures in each cluster of 60.4 ± 19.3 (range 25‒104) and a mean number of seizures per day of 20.2 ± 2.8 (range 14‒25) at the peak. The mean duration of the seizure clusters was 7.3 ± 1.0 days (range 6‒9 days), and the mean inter-cluster interval was 15.6 ± 4.3 days (range 11‒24 days).

**Figure 2:**
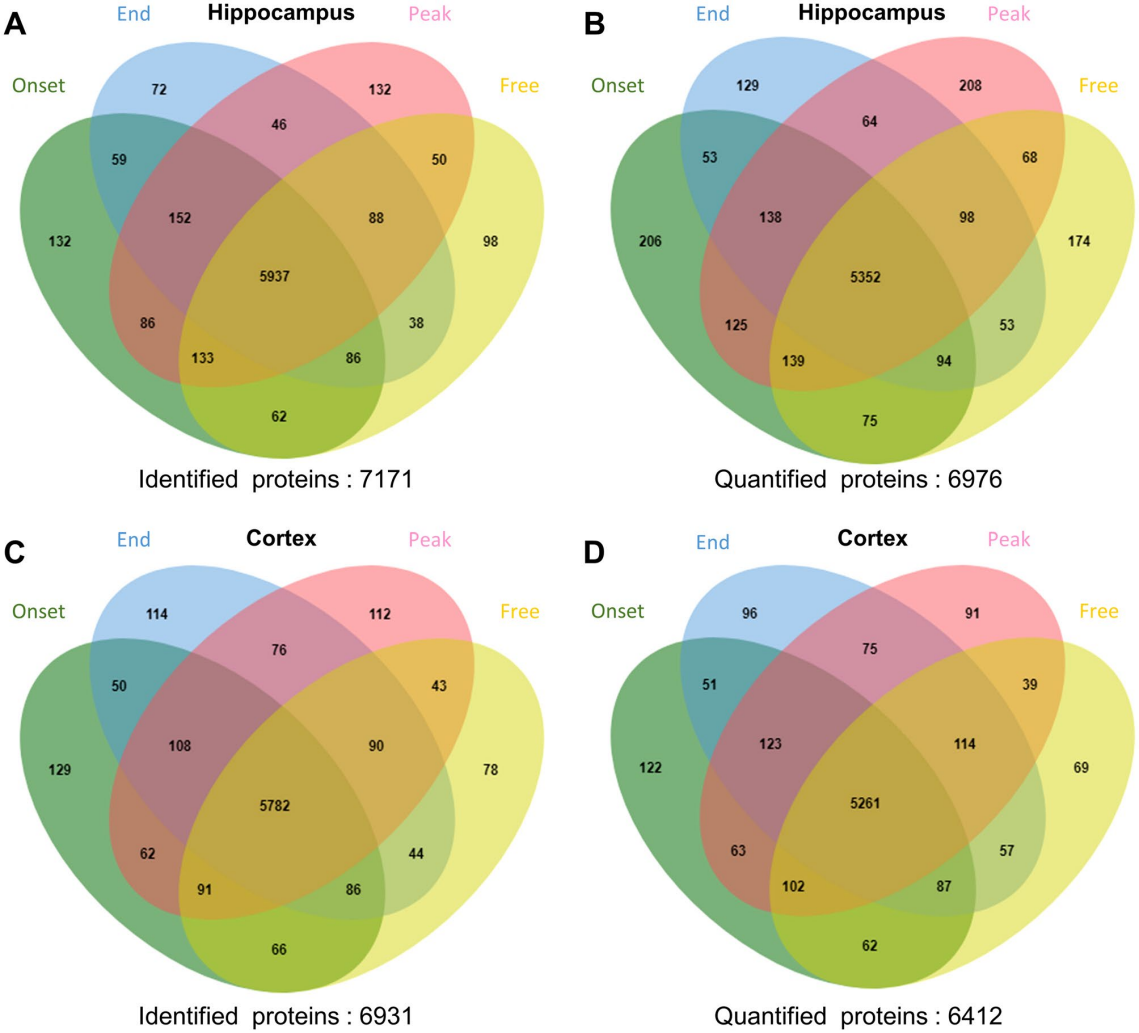
Venn diagram of the protein identification and quantification. Panels **(a**, **b)** show the numbers of identified (**a**) and quantified (**b**) proteins at each time point, including the onset of the seizure cluster (onset), peak of the seizure cluster (peak), end of the seizure cluster (end), and mid seizure-free period (free) in the hippocampus. Panels **c** and **d** display the numbers of identified (**c**) and quantified (**d**) proteins at each time point in the cortex.

**Figure 3:**
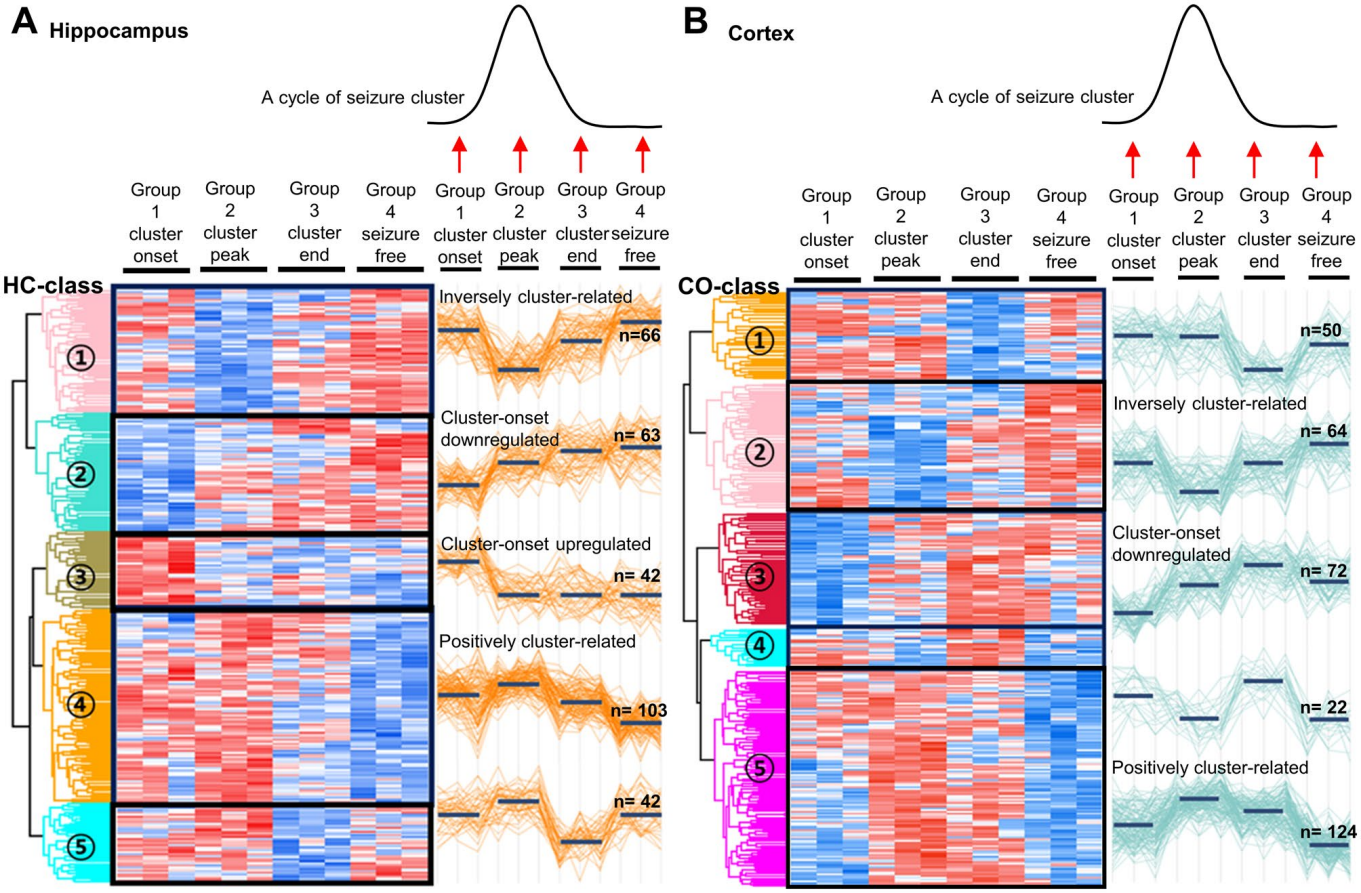
Hierarchical clustering and classification of the differentially expressed proteins. In panel **(a)**, differentially expressed proteins (DEPs) in the hippocampus are classified into 5 classes, according to their expression pattern in different time points during a seizure cluster. Hippocampal class 1 (HC-class 1) exhibits the lowest expression level at the peak of a seizure cluster and gradually increases the expression toward the end of a cluster and the seizure-free period, and designated as ‘inversely cluster-related DEPs’. Accordingly, HC-class 2 was designated as cluster-onset downregulated DEPs, HC-class 3 as cluster-onset upregulated DEPs, and HC-class 4 as positively cluster-related DEPs. HC-class 5 does not exhibit a specific cluster-related expression pattern. In panel **(b)**, cortex class 2 (CO-class 2) was designated as inversely cluster-related DEPs, CO-class 3 as cluster-onset downregulated DEPs, and CO-class 5 as positively cluster-related DEPs. CO-class1 and CO-class 4 do not exhibit a clinically relevant cluster-related expression pattern.

In the proteomic analysis, a total of 7171 proteins were identified and 6976 proteins were quantified in the hippocampus, and a total of 6931 proteins were identified and 6412 proteins were quantified in the cortex (Fig. 2, full expression profiles in Supplementary Dataset 1). For each brain sample, the mean values of normalized protein expression level in the hippocampus and in the cortex did not show any significant correlation with the time from SE to brain tissue acquisition or the number of seizures prior to brain tissue acquisition (Supplemental Table 1), or an inter-group difference (Supplemental Table 2). In the hippocampus, analysis of variance (ANOVA) analysis returned 317 differentially expressed proteins (DEPs) which were categorized into 5 classes by hierarchical clustering, and in the cortex, 332 DEPs were identified and categorized into 5 classes (Fig. 3, full list of DEPs in Supplementary Dataset 2).

### DEP classes in the hippocampus and in the cortex

For the hippocampal DEP classes (HC-classes), HC-class 1 (66 DEPs) exhibited the lowest expression level at the peak of a seizure cluster and gradually increased the expression toward the end of a cluster and the seizure-free period. HC-class 2 (63 DEPs) showed the lowest expression level at the onset of a seizure cluster and showed gradually elevated expression levels toward the seizure-free period. HC-class 3 (42 DEPs) showed the highest expression level at the onset of a seizure cluster and returned to low expression levels at all other time points. HC-class 4 (103 DEPs) exhibited the highest level at the peak of a seizure cluster with a gradually decreasing pattern, whereas HC-class 5 (42 DEPs) did not exhibit a specific cluster-related expression pattern (Fig. 3).

We hypothesized that HC-classes 1 and 4 might represent proteins pathogenetically involved in the inhibition and activation of seizure clusters, respectively, or at least reflect the consequences of clustered seizures^26,27^. However, the protein expression profiles in classes 1/4, which gradually increased/decreased after the cluster peak and were still low/high at the cluster end, could not be sufficiently explained by the former hypothesis that those DEPs directly contribute to the pathogenesis of a seizure cluster. Therefore, we designated HC-classes 1 and 4 as inversely cluster-related DEPs and positively cluster-related DEPs, respectively. However, as HC-classes 2 and 3 were specifically suppressed/activated at the cluster onset, we inferred those classes might have pathogenetic roles in ictogenesis and designated HC-class 2 as cluster-onset downregulated DEPs and HC-class 3 as cluster-onset upregulated DEPs.

For the cortex DEP classes (CO-classes), CO-class 1 (50 DEPs) exhibited the lowest expression level at the end of a seizure cluster, and CO-class 2 (72 DEPs) showed the lowest expression level at the peak of a seizure cluster with a gradual increase toward the seizure-free period. CO-class 3 (64 DEPs) showed the lowest expression level at the onset of a seizure cluster, gradually increased the expression level toward the end of a seizure cluster, and had a decreased level at the seizure-free period, whereas CO-class 5 (124 DEPs) showed the highest expression level at the peak of a seizure cluster with gradual decrease toward the seizure-free period. CO-Class 4 (22 DEPs) did not exhibit a specific cluster-related expression pattern (Fig. 3). As the expression patterns of CO-classes 2 and 5 were similar to those of the HC-classes 1 and 4, those classes were designated as inversely/positively cluster-related DEPs, respectively. Additionally, as CO-class 3 and HC-class 2 exhibited similar expression patterns, CO-class 3 proteins were designated as cluster-onset downregulated DEPs.

### Specificity analysis of the DEPs in the HC-classes

**Figure 4:**
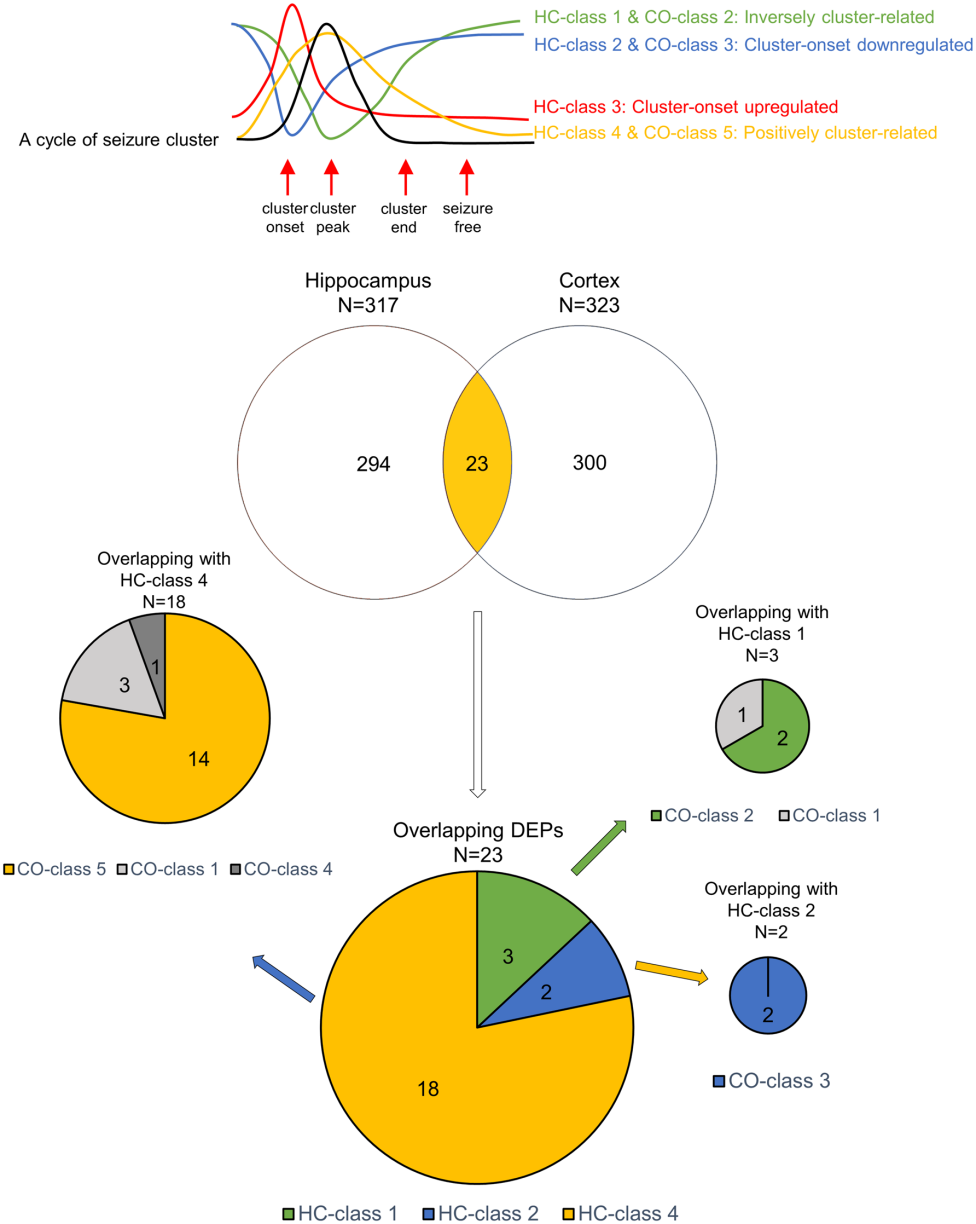
Protein overlap between hippocampal and cortical differentially expressed protein classes. A total of 23/317 (7.3%) differentially expressed proteins (DEPs) in the hippocampus show an overlap with DEPs in the cortex, including 3 of hippocampal class (HC-class) 1, 2 of HC-class 2, and 18 HC-class 4 DEPs. HC-class 3 and HC-class 5 DEPs do not show any overlap with any of the DEPs in the cortex. DEP overlap in each HC-classes with CO-classes are also demonstrated. Expression patterns of each cluster of DEPs are demonstrated in the upper panel. DEP classes with same expression pattern between the hippocampus and the cortex are indicated with a same color.

A total of 23/317 (7.3%) DEPs in the hippocampus overlapped with the DEPs in the cortex, including 3/66 (4.5%) of HC-class 1, 2/63 (3.2%) of HC-class 2, and 18/103 (17.5%) HC-class 4 DEPs. HC-class 3 and 5 DEPs did not show any overlap with any of the DEPs in the cortex. In HC-class 4, 14/18 (77.8%) overlapping DEPs were in CO-class 5, 3 (16.7%) were in CO-class 1, and the remaining 1 (5.6%) was in CO-class 4. In HC-class 1, 2/3 (66.7%) overlapping DEPs were in CO-class 2, and the remaining 1 (33.3%) was in CO-class 1. All (2/2, 100.0%) overlapping DEPs in HC-class 2 were in CO-class 3. Considering that HC-class 4 and CO-class 5 were commonly designated as positively cluster-related DEPs, this overlap indicates that the DEPs in HC-class 4 might represent common biological consequences of clustered seizure activities spreading across the brain. However, HC-class 3 exhibited no DEP overlap with the cortex, which indicates that a high spatial specificity hippocampal protein regulation is involved in the pathomechanism underlying the development of a seizure cluster (Fig. 4).

### Gene ontology and pathway analysis

Gene ontology (GO) analysis was performed to evaluate the connection of each DEP cluster with biological processes. The most commonly involved biological processes were in HC-class 1 cellular respiration and metabolic processes, in HC-class 2 signaling pathway, membrane transport, and energy metabolism, in HC-class 3 axonogenesis, synaptic vesicle assembly, and neuronal projection or filopodium morphogenesis, and in HC-class 4 biosynthesis, subunit assembly, metabolic processes, and neuronal differentiation (Table 1). In the pathway analyses, the most frequently associated pathways were the citric acid (TCA) cycle, carbon metabolism, and extracellular matrix (ECM)-receptor interaction in HC-class 1, TCA cycle and GABAergic synapse in HC-class 2, GABAergic synapse, glutamatergic synapse, and inflammatory mediator regulation of transient receptor potential (TRP) channels in HC-class 3, and ribosome and proteasome in HC-class 4 (Table 2, full GO and pathway analysis results in Supplementary Dataset 3). We inferred that the frequent occurrence of cellular respiration and metabolic processes in HC-class 1 reflects the disrupted homeostatic cell metabolism caused by clustered seizures^26^. Similarly, enrichment for biosynthesis, metabolic processes, and neuronal differentiation in HC-class 4 also might reflect the seizure cluster-related activation of those processes in the neuronal system^26,27^.

**Table 1:** Top five enriched Gene ontology biological processes for each differentially expressed protein (DEP) classes in the hippocampus. For the five hippocampal (HC)-DEP classes, top five enriched Gene ontology biological processes are summarized.

Term	Fold enrichment	*P* value	FDR
**HC-class 1: inversely cluster-related DEPs**			
Dicarboxylic acid metabolic process	< 0.001	< 0.001**	< 0.001
Organic acid metabolic process	< 0.001	< 0.001**	< 0.001
Carboxylic acid metabolic process	0.001	< 0.001**	0.002
Oxoacid metabolic process	0.001	< 0.001**	0.003
Oxidation–reduction process	0.004	< 0.001**	0.014
**HC-class 2: cluster-onset downregulated DEPs**			
Energy derivation by oxidation of organic compounds	0.892	0.001**	1.794
Generation of precursor metabolites and energy	0.991	0.004**	7.461
Regulation of G-protein coupled receptor protein signaling pathway	0.969	0.005**	8.168
Central nervous system neuron axonogenesis	0.953	0.006**	9.464
Regulation of cation transmembrane transport	0.943	0.007**	11.045
**HC-class 3: cluster-onset upregulated DEPs**			
Regulation of cell morphogenesis	0.717	0.001**	1.565
Cellular protein complex assembly	0.894	0.003**	5.447
Regulation of filopodium assembly	0.839	0.004**	6.616
Filopodium assembly	0.839	0.004**	6.616
Neuron projection morphogenesis	0.827	0.005**	8.388
**HC-class 4: positively cluster-related DEPs**			
Organonitrogen compound biosynthetic process	< 0.001	< 0.001**	< 0.001
Translation	0.004	< 0.001**	0.006
Peptide biosynthetic process	0.003	< 0.001**	0.008
Amide biosynthetic process	0.008	< 0.001**	0.025
Peptide metabolic process	0.015	< 0.001**	0.062
**HC-class 5**			
Establishment of protein localization to organelle	0.707	0.001**	1.286
Wnt signaling pathway	0.605	0.001**	1.942
Cell–cell signaling by wnt	0.465	0.001**	1.964
Cell surface receptor signaling pathway involved in cell–cell signaling	0.634	0.003**	4.157
Apoptotic process	0.559	0.003**	4.232

*FDR* false discovery rate, *HC* hippocampus, *DEP* differentially expressed proteins. ** *P* < 0.01.

**Table 2:** Top five enriched pathways for each differentially expressed protein classes in the hippocampus. For the five hippocampal (HC)-DEP classes, top five enriched pathways are summarized.

Term	Fold enrichment	*P* value	FDR
**HC-class 1: inversely cluster-related DEPs**			
Citrate cycle (TCA cycle)	0.001	< 0.001**	0.016
Carbon metabolism	0.001	< 0.001**	0.017
ECM-receptor interaction	0.021	0.001**	0.831
Biosynthesis of antibiotics	0.066	0.003**	3.474
Cardiac muscle contraction	0.091	0.006**	6.036
**HC-class 2: cluster-onset downregulated DEPs**			
Citrate cycle (TCA cycle)	0.540	0.007**	7.409
Influenza A	0.804	0.028*	27.617
GABAergic synapse	0.826	0.045*	40.487
Biosynthesis of antibiotics	0.766	0.049*	43.739
**HC-class 3: cluster-onset upregulated DEPs**			
GABAergic synapse	0.158	0.002**	1.773
Glutamatergic synapse	0.173	0.004**	3.895
Inflammatory mediator regulation of TRP channels	0.152	0.005**	5.010
Vascular smooth muscle contraction	0.720	0.046*	41.149
**HC-class 4: positively cluster-related DEPs**			
mmu03010: Ribosome	< 0.001	< 0.001**	0.005
mmu03050: Proteasome	0.880	0.035*	33.769
**HC-class 5**			
Adherens junction	0.758	0.018*	17.173
Ribosome biogenesis in eukaryotes	0.605	0.023*	21.873

*FDR* false discovery rate, *HC* hippocampus, *DEP* differentially expressed proteins. * *P* < 0.05 and ** *P* < 0.01.

### Hippocampal expression of key proteins of each DEP class in HC-class 2 and HC-class 3

As we inferred that DEPs in HC-class 2 and HC-class 3 might be pathogenically relevant to the cluster-onset downregulated and cluster-onset upregulated processes, respectively, we searched for the top five proteins most abundantly involved in the enriched biological processes of HC-classes 2 and 3, which returned PRKCA, EZR, S100B, SLC8A1, and SNCA for HC-class 2 and DNM3, CADPS, SYT2, SYT6, and FSCN1 for HC-class 3.

**Figure 5:**
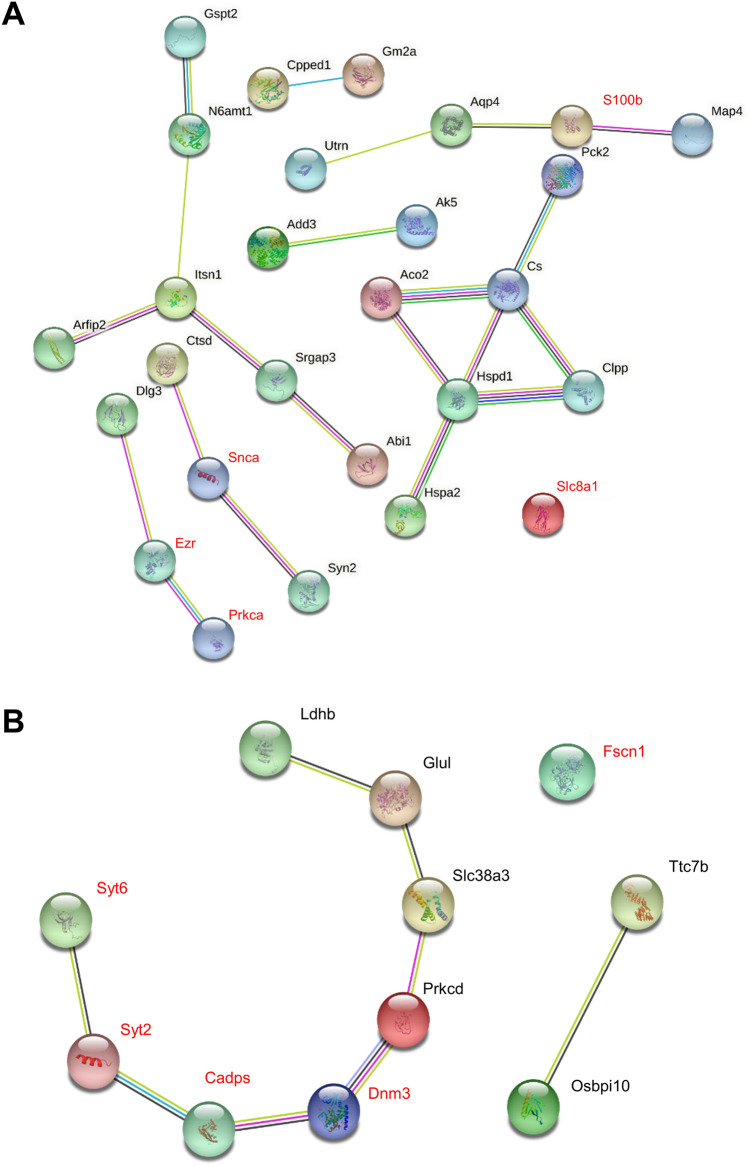
STRING analysis of the differentially expressed proteins in hippocampal (HC)-classes 2 and 3. In the STRING analysis of the differentially expressed proteins (DEPs) in HC-class 2 (**a**), the key DEPs PRKCA, EZR, S100B, SLC8A1, and SNCA are not interconnected by protein–protein interactions (marked in red). By contrast, the STRING analysis of the DEPs in HC-class 3 (**b**) reveals the interconnection among the key DEPs (DNM3, CADPS, SYT2, and SYT6) except for FSCN1 (marked in red).

We designated those proteins as the key DEPs in each class and screened the literature regarding their functions and potential roles in ictogenesis. For HC-class 3, the ictogenesis-related functions of the key DEPs were homogeneous including synaptic vesicle assembly, exocytosis, and neurotrophin release (DNM3, CADPS, SYT2, and SYT6) and neurite assembly and neuronal projection (DNM3, SYT2, and FSCN1)^28–35^. By contrast, the ictogenesis-related functions of the key DEPs in HC-class 2 were heterogeneous including synaptic plasticity by trafficking of *N*-Methyl-D-Aspartate receptors (PRKCA)^36^, cytoskeleton-cell membrane interaction (EZR)^37^, cell growth and differentiation (EZR and S100B)^37,38^, enhancement of cell survival and proliferation, as well as anti-apoptotic signaling in neuronal cells (S100B)^38^, increased cellular calcium uptake (S100B)^39^, calcium removal from cells via the sodium-calcium exchanger (SLC8A1)^40^, and production of alpha-synuclein (SNCA; Table 3)^41^. In STRING analyses for protein–protein interactions, DEPs in HC-class 3 exhibited interconnection among the key DEPs except for FSCN1, whereas the key DEPs in HC-class 2 did not (Fig. 5).

**Table 3:** Seizure-related function of the key differentially expressed proteins in classes 2 and 3.

Protein	Abbreviation	Seizure-related function
**HC-class 2: cluster-onset downregulated DEPs**		
Protein kinase C alpha	Prkca	Synaptic plasticity by trafficking of N-Methyl-D-Aspartate receptors
Ezrin	Ezr	Cytoskeleton-membrane interference, cell growth, migration, and differentiation
Calcium-binding protein B	S100b	Promote cell survival, differentiation, neurite outgrowth, anti-apoptosis of neurons, stimulate Ca^2+^ uptake in neurons and astrocytes
Solute carrier family 8 member A1	Slc8a1	Sodium-calcium exchanger that removes calcium from cells
Alpha-synuclein	Snca	Production of alpha-synuclein
**HC-class 3: cluster-onset upregulated DEPs**		
Dynamin-3	Dnm3	Synaptic vesicle assembly, dendritic spine morphogenesis and remodeling
Calcium-dependent secretion activator	Cadps	Synaptic vesicle assembly, Ca^2+^ dependent neurotransmitter and neurotrophin release in synapse
Synaptotagmin-2	Syt2	Synaptic vesicle assembly, Ca^2+^ dependent exocytosis of synaptic vesicles, neurotransmitter release
Synaptotagmin-6	Syt6	Ca^2+^ dependent exocytosis of synaptic vesicles, neurotransmitter release
Fascin-1	Fscn1	Dynamic projections of neuronal dendrites, regulate processes of axonal motility and synaptic function

*HC* hippocampus, *DEP* differentially expressed proteins.

## Discussion

This study performed a proteomic analysis in the hippocampus of a murine pilocarpine-induced chronic epilepsy model and demonstrated classes of differentially expressed proteins with distinct expression patterns during a seizure cluster, as well as their pathogenetic functions and involved biological processes. Among the five identified hippocampal DEP classes, HC-class 3 exhibited a specific expression pattern that was not observed in the cortex, although the expression patterns of HC-classes 1, 2, and 4 resembled those of CO-classes 2, 3, and 5, respectively. Additionally, DEPs in HC-class 3 were specific to the hippocampus, whereas HC-class 4 exhibited a significant overlap with CO-class 5. Especially, HC-class 3 showed a specific pattern suggesting a pro-ictogenesis function of the proteins in this class. DEPs in HC-class 3 were involved in axonogenesis, synaptic vesicle assembly, and neuronal projection, indicating a pathomechanistic role of these processes in ictogenesis^6,42^. Five key proteins in HC-class 3 had abundant involvement in the predominant associated biological processes and were interconnected. HC-class 2 showed an expression pattern suggesting an anti-ictogenic function. However, the ictogenesis-related functions of the key proteins were heterogeneous, and the key proteins in HC-class 2 were less interconnected.

Epileptogenesis is a chronic indolent process that may develop over several years from the initial insult or genetic predisposition. In most cases, the epileptogenic process has already progressed or is completed at the time of diagnosis, which is a major obstacle to the clinical investigation of anti-epileptogenesis strategies^43^. Seizure clustering is a common phenomenon in chronic epilepsy, even in patients with intractable epilepsy and frequent seizure attacks, and associated with increased risk of SE, seizure-related complications, and hospitalization. Therefore, targeting ictogenesis and related seizure clustering might provide a novel and more clinically applicable strategy for treating chronic epilepsy over the currently available anti-epileptic drugs that suppress seizure activity itself by targeting ion channels, neurotransmitters, or synaptic vesicles^4,6,42^.

In this regard, proteomic analysis has advantages in identifying key proteins of ictogenesis by evaluating the site-specific dynamic changes of the molecular processes. Previous studies performed proteomic analyses on epileptogenesis. A study using a rat model of chronic epilepsy demonstrated that proteins related to inflammatory processes including toll-like receptor signaling, pro-inflammatory cytokines, heat shock protein regulation, transforming growth factor-beta signaling, and leukocyte migration were upregulated during epileptogenesis^9^. Another study demonstrated that pathways linked with neuronal death, ECM-to-ECM interaction, ECM-to-cell interaction, and angiogenesis were associated with the epileptogenic process^8^. However, ours is the first study using proteomic analysis focused on ictogenesis processes in a chronic epilepsy model with clustering seizure pattern.

Although this study did not directly investigated the mechanism of the DEPs on ictogenesis, biological processes involving HC-class 3 DEPs might reflect the major constitutes of the ictogenesis process, although the exact pathomechanism is still enigmatic. Computational modeling and in vitro analyses using hippocampal slices have reported that transition from a seizure-free status to seizure is a progressive process rather than a suddenly triggered phenomenon, that is characterized by a gradual increase in excitability, reduction of stability, and loss of resilience in the neuronal network^7,44,45^. The molecular changes underlying ictogenesis, characterized by interictal discharges with increasing duration and a higher number of recruited neurons, cause high-frequency firing of interneurons resulting in increased Cl^−^ levels in postsynaptic cells, leading to the reduction or even reversal of GABAergic inhibitory signals. This is accompanied by increased neuronal volumes, changes in neuronal projection, and alterations in ion channel distribution to promote intraneuronal Cl^−^ accumulation, downregulation of K^+^ channels in astrocytes leading to increases in extracellular K^+^, and increased release and reduced clearance of glutamate by neurons and astrocytes^6,7,42^. These gradually intensified processes of the neuronal network toward a seizure-prone state with high excitability and synchrony might have been reflected by the enriched biological processes and pathways of HC-class 3 DEPs, which are axonogenesis, synaptic vesicle assembly, neuronal projection, and changes involving GABAergic and glutamatergic synapses^6,7,42,44^. In this regard, the five key proteins in HC-class 3, DNM3, CADPS, SYT2, SYT6, and FSCN1, might serve as potential biomarkers for seizure prediction or a target for anti-seizure treatment in chronic epilepsy, as these proteins were abundantly involved in biological processes relevant to ictogenesis.

Although not comprehensively discussed, HC-classes 1, 2, and 4 might also reflect site- and phase-specific biological changes during a seizure cluster. HC-class 2 proteins are inferred to involve anti-ictogenesis processes by regulating calcium signaling, membrane interaction and transport, synaptic plasticity, and neuronal growth and differentiation^36–38,40,41^. HC-class 1 proteins might reflect diminished cellular respiration and metabolic processes due to clustered seizures^26^, and HC-class 4 proteins represent common consequences of clustered seizures spreading across the brain, namely increased biosynthesis and cell metabolism, ribosome and proteasome activity, and neuronal differentiation^26,27^. Therefore, this study also provides a database for the site and time point of specific molecular changes during initiation, progression, and cessation of clustered seizures and provides a basis for elucidating the pathomechanism of ictogenesis and seizure clustering. However, as only four time-points during a seizure cluster, this study data does not fully elucidate the proteomic pathomechanism of seizure clustering. More detailed protein expression data with higher temporal resolution might be necessary, especially during the seizure-free period, to fully investigate the role of DEPs in the pathomechanism of seizure clustering.

This study has some limitations to be addressed in future work. First, as this study data were derived from a large-scale proteomic analysis, their differential expression was not validated by confirmative analyses. Second, as the sacrifice time point during a seizure cluster was determined according to the previous seizure clusters of a mouse, inter-cluster variation of the cluster duration, severity, and period might be potential sources of bias. Third, as this study did not directly evaluated the causal relation of DEPs on ictogenesis and seizure clustering, further investigations validating the cell-specific expression patterns of the identified key proteins, their role in ictogenesis, and the seizure-preventing effects by regulating these proteins might be required. Fourth, this study did not include a control group. Although this study focused on the proteins with significant change during a seizure cluster cycle, comparison of protein expression with non-epileptic mice might provide useful information on the effect of chronic epilepsy on protein regulation. Additionally, only 3 mice were selected from groups 1, 3, and 4, which might possibly be a source of selection bias.

In conclusion, these study data provide comprehensive information about the spatial and temporal regulation of protein expression during a seizure cluster. In particular, the HC-class 3 proteins, with elevated expression levels in the hippocampus specifically at the initiation of a seizure cluster, provide novel insights regarding the biological processes involved in ictogenesis. The key proteins of HC-class 3 might serve as biomarkers for predicting seizure occurrence or as treatment targets for reducing seizure attacks in patients with chronic epilepsy.

## Supplementary Information

Supplementary Information 1.

Supplementary Information 2.

Supplementary Information 3.

Supplementary Information 4.

## Data Availability

The datasets generated or analyzed during the current study are available in Supplemental Datasets.
