# Supplementary material for: Proteins related to ictogenesis and seizure clustering in chronic epilepsy
Source: Sci Rep. 2021 Nov 2;11:21508. doi: 10.1038/s41598-021-00956-6 (PMC8563854; doi:10.1038/s41598-021-00956-6)
Supplement: Supplementary file 1 — Supplementary Information 1. [file 41598_2021_956_MOESM1_ESM.docx]

Supplemental Materials

**Proteins related to ictogenesis and seizure clustering in chronic epilepsy**

**List of Supplements:**

**Supplemental Table 1.** Correlation analyses between the mean values of normalized protein expression level in the hippocampus and in the cortex with the time from SE or the number of seizures to brain tissue acquisition

|  | **HC normalized protein** | **Cortex normalized protein** | **Number of seizure prior to scarification** | **Duration from SE to scarification (days)** |
| --- | --- | --- | --- | --- |
| **Total mouse (n=12)** |  |  |  |  |
| HC normalized protein | - | r=-0.377, *P*=0.228 | r=0.221, *P*=0.490 | r=0.080, *P*=0.804 |
| Cortex normalized protein | r=-0.377, *P*=0.228 | - | r=-0.227, *P*=0.479 | r=0.343, *P*=0.275 |
| **Group 1 (n=3)** |  |  |  |  |
| HC normalized protein | - | r=0.228, *P*=0.853 | r=-0.194, *P*=0.876 | r=-0.912, *P*=0.268 |
| Cortex normalized protein | r=0.228, *P*=0.853 | - | r=0.911, *P*=0.271 | r=-0.190, *P*=0.878 |
| **Group 2 (n=3)** |  |  |  |  |
| HC normalized protein | - | r=-0.918, *P*=0.259 | r=0.717, *P*=0.491 | r=0.575, *P*=0.610 |
| Cortex normalized protein | r=-0.918, *P*=0.259 | - | r=-0.935, *P*=0.232 | r=-0.204, *P*=0.869 |
| **Group 3 (n=3)** |  |  |  |  |
| HC normalized protein | - | r=-0.402, *P*=0.737 | r=0.950, *P*=0.202 | r=-0.279, *P*=0.820 |
| Cortex normalized protein | r=-0.402, *P*=0.737 | - | r=-0.668, *P*=0.534 | r=0.891, *P*=0.438 |
| **Group 4 (n=3)** |  |  |  |  |
| HC normalized protein | - | r=-0.488, *P*=0.676 | r=0.627, *P*=0.569 | r=0.982, *P*=0.122 |
| Cortex normalized protein | r=-0.488, *P*=0.676 | - | r=-0.986, *P*=0.107 | r=-0.645, *P*=0.554 |

HC: hippocampus and SE: status epilepticus

**Supplemental Table 2.** Inter-group comparison of normalized protein expression level

|  | **Total mouse (n=12)** | **Group 1 (n=3)** | **Group 2 (n=3)** | **Group 3 (n=3)** | **Group 4 (n=3)** | ***P*** |
| --- | --- | --- | --- | --- | --- | --- |
| **HC normalized protein** | 26.5±0.0 | 26.5±0.0 | 26.6±0.0 | 26.5±0.1 | 26.5±0.0 | 0.791 |
| **Cortex normalized protein** | 27.3±0.0 | 27.3±0.0 | 27.4±0.0 | 27.3±0.1 | 27.3±0.0 | 0.877 |

HC: hippocampus
